# Rasch analysis of the participation scale (P-scale): usefulness of the P-scale to a rehabilitation services network

**DOI:** 10.1186/s12889-017-4945-9

**Published:** 2017-12-08

**Authors:** Mariana Angélica Peixoto Souza, Wendy Jane Coster, Marisa Cotta Mancini, Fabiana Caetano Martins Silva Dutra, Jessica Kramer, Rosana Ferreira Sampaio

**Affiliations:** 1 Graduate Program in Rehabilitation Sciences – School of Physical Education, Physical Therapy and Occupational Therapy, Universidade Federal de Minas Gerais, Belo Horizonte, Brazil; 2 Department of Occupational Therapy – Sargent College, Boston University, Boston, USA; 3 Department of Occupational Therapy – School of Physical Education, Physical Therapy and Occupational Therapy, Universidade Federal de Minas Gerais, Belo Horizonte, Brazil; 4 Department of Occupational Therapy – Centro Educacional, Universidade Federal do Triângulo Mineiro, Uberaba, Brazil; 5 Department of Physical Therapy, School of Physical Education, Physical Therapy and Occupational Therapy, Universidade Federal de Minas Gerais, Avenida Antonio Carlos, 6627, Campus Universitário, Pampulha, Belo Horizonte, MG ZIP Code: 31270-401 Brazil

**Keywords:** Outcome assessment (health care), Social participation, Rehabilitation

## Abstract

**Background:**

A person’s participation is acknowledged as an important outcome of the rehabilitation process. The Participation Scale (P-Scale) is an instrument that was designed to assess the participation of individuals with a health condition or disability. The scale was developed in an effort to better describe the participation of people living in middle-income and low-income countries. The aim of this study was to use Rasch analysis to examine whether the Participation Scale is suitable to assess the perceived ability to take part in participation situations by patients with diverse levels of function.

**Methods:**

The sample was comprised by 302 patients from a public rehabilitation services network. Participants had orthopaedic or neurological health conditions, were at least 18 years old, and completed the Participation Scale. Rasch analysis was conducted using the Winsteps software.

**Results:**

The mean age of all participants was 45.5 years (standard deviation = 14.4), 52% were male, 86% had orthopaedic conditions, and 52% had chronic symptoms. Rasch analysis was performed using a dichotomous rating scale, and only one item showed misfit. Dimensionality analysis supported the existence of only one Rasch dimension. The person separation index was 1.51, and the item separation index was 6.38. Items N2 and N14 showed Differential Item Functioning between men and women. Items N6 and N12 showed Differential Item Functioning between acute and chronic conditions. The item difficulty range was −1.78 to 2.09 logits, while the sample ability range was −2.41 to 4.61 logits.

**Conclusions:**

The P-Scale was found to be useful as a screening tool for participation problems reported by patients in a rehabilitation context, despite some issues that should be addressed to further improve the scale.

## Background

Since the World Health Organization (WHO) published the International Classification of Functioning, Disability and Health (ICF), interest in better understanding and assessing the “participation” construct has been a frequent topic in rehabilitation literature. In the ICF, participation is defined as “involvement in life situations”, or the result of a complex relationship between a person, his or her health condition, and the environment [1, 2]. A person’s participation is acknowledged as an important outcome of the rehabilitation process, even though several researchers have identified the need for a better conceptualization of the participation construct in order to build better measurement tools [3–6].

The rehabilitation process encompasses a set of procedures aiming to assist individuals who experience or are likely to experience disability to achieve and maintain optimal functioning in interacting with their environments [7]. Thus, information regarding a person’s functioning, including participation, is necessary to guide rehabilitation planning and assess the impact of intervention. Recent systematic reviews have identified a variety of participation measures available in the literature [8, 9]. However, most of these participation tools were developed in high-income countries and may not be suitable to represent the experiences of those living in less developed societies [10, 11].

In an effort to better describe the participation of people living in middle-income and low-income countries, van Brakel et al. (2006) proposed the Participation Scale (P-Scale) – an instrument that was designed to assess the participation of individuals with a health condition or disability, especially conditions associated with stigma and discrimination [10]. The P-Scale aims to quantify the restrictions perceived by the individual in eight of the nine major areas of life defined by the ICF: learning and applying knowledge; communication; personal care; mobility; domestic life; interpersonal interactions and relationships; major areas of life; and community, social and civic life [10]. An innovative characteristic of the scale is that the individuals are asked to compare themselves with a real or hypothetical “peer” – that is, someone who is similar to them in all respects, except for illness or disability. This comparison was proposed to allow the representation of the roles and expectations for participation in different social and cultural contexts [10]. These special features indicate that the P-scale might be useful to assess clients’ participation restrictions in diverse life situations.

In Brazil, the public rehabilitation services are structured in an integrated network, organized with multidisciplinary teams across three levels of care (basic, specialized, and hospital) [12]. In addition to the diversity of professionals and services, the rehabilitation networks have to address diverse patient profiles because the services provide assistance to people of a wide variety of health conditions, socio-demographic backgrounds, and functional needs [12–14]. In this clinical context, information about patients’ function, including participation restrictions, may help service planning and better direct investments in rehabilitation [15, 16]. Therefore, it would be helpful to investigate the P-Scale properties and its suitability to support rehabilitation services in the country.

In previous validation studies, the P-Scale showed good psychometrics properties and was found to be valid for use in several different health conditions and cultural environments [10, 11, 17, 18]. All these studies, however, used Classical Test Theory (CTT) procedures, and, to the best of our knowledge, there is no study that assessed the P-Scale properties using Rasch analysis. Classical Test Theory has a number of limitations, including sample dependency (the item and scale statistics apply only to a specific group of respondents) and the assumption of item equivalence (individual items are treated as being equally difficult) [19, 20].

On the other hand, Rasch analysis transforms ordinal data (i.e., ratings with non-equal intervals) into linear measures with equal-interval units called logits, which are used to describe the measures of both individuals and items [21]. The transformation of raw scores into an abstract linear continuum of ability (for individuals) and difficulty (for items) allows one to predict the likelihood of a person choosing, for example, “yes or no” on a specific functional item [22]. Thus, one is able to identify the location of each item on a continuum of ability and compare where the person’s level of ability is located on the same continuum.

Once the person and item measures are described using the same “logit” unit, Rasch analysis allows for the comparison of a person to other individuals, one item to other items, and individuals to items [21]. Furthermore, the Rasch model can be used to build new scales, to suggest improvements to existing scales and to estimate the stability of item difficulty estimates among different groups, thus allowing for comparisons of homogeneous measures [22]. The aims of this study were to use Rasch analysis for the following:

- to assess the P-Scale items in terms of their item and person fit, dimensionality, item difficulty, reliability, and Differential Item Functioning (based on gender and duration of the present symptoms);
- and to examine whether the Brazilian-Portuguese version of P-Scale is suitable to assess the perceived ability to take part in participation situations by patients in a rehabilitation services network who have diverse levels of function.

## Methods

### Participants and procedures

Data for this study comprised two datasets: the first one was collected from January to December 2010 (*n* = 216) [23]; and the second one was collected from April to June 2014 (*n* = 86). These two datasets together constituted a convenience sample of 302 patients who were seeking treatment or were in treatment in one basic-care or two specialized-care services that are part of a public rehabilitation network in Brazil. To be eligible for inclusion, participants had to have an orthopaedic or a neurological health condition (acute or chronic), be at least 18 years old, and be able to understand and answer the interview questions. There was no upper age limit or other limitation in the type of health condition. All participants were informed about the study and gave informed consent. Then, the participants were interviewed using a socio-demographic questionnaire and the P-Scale. The Ethics Committee from Universidade Federal de Minas Gerais, Brazil, approved the study (n. 426.982).

The interviews were conducted by one of the authors (FCMSD or MAPS). Prior to data collection, both interviewers did a careful reading of the P-Scale manual, and any doubts or issues were discussed with a third researcher (RFS). Additional questions not solved among the researchers were emailed to the P-Scale author (Wim van Brakel). In the 2010 dataset, the interviews were conducted using a former version of the scale [10], while in the 2014 dataset, the more recent P-Scale available was used [24]. These two versions are essentially similar, with the main distinctions being the sequence of the items and the replacement of one item (i.e., Item *N16 – In home, are the eating utensils you use kept with those used by the rest of the household?* in the former version was replaced by Item *N10 – Do you have the same opportunities as your peers to start or maintain a long-term relationship with a life partner?* in the latest version). For this study we used only the items present in both versions.

### Participation scale

The P-Scale has 18 items, in which the person is asked to respond whether they perceive their level of participation as equal to their “peer” in each of the situations described by the scale items. If the person considers that his/her level of participation is lower than that of his/her peer, representing a possible restriction to participation, he/she is also asked to indicate to what degree this is a problem in his/her daily routine [10]. The individual’s score on each item can be “no problem” = 1, “Small” = 2, “Medium” = 3, “Large” = 5, or 0 (zero) if the individual does not consider his/her participation less than that of his/her peer. To obtain the total score, values attributed to each item are added. The P-scale total score varies between 0 (zero) and 90, where 0 = “no restrictions on participation” and 90 = “complete restriction in participation” [10].

Van Brakel et al. (2006) performed an initial validation study, in which the P-Scale showed a Cronbach’s alpha of 0.92, an intra-class correlation coefficient for intra-interviewer stability of 0.83 and an inter-interviewer reliability of 0.80; 90% of variability was explained in the first factor in Factor analysis [10]. Other studies that also conducted Factor analyses found a better fit to the two-factor model (“work-related participation” and “general participation”) [11, 25], although these factors can also be part of an unidimensional scale because they were strongly correlated with each other [25].

### Data entry and statistical analysis

The Statistical Package for Social Sciences (SPSS) – v.16 was used for data entry, dataset management, and descriptive statistics. After the data entry, the dataset was double-checked by the researchers FCMSD or MAPS. The final dataset did not have missing data. Rasch analysis was conducted using the Winsteps software – v.3.81.0.

Individual item and person fit were analysed using Infit and Outfit statistics to indicate how well data conformed to the Rasch model. For each one of these fit statistics, Winsteps provides Mean Square (MNSQ) and Z-Standardized Scores (ZSTD) [21]. As a general rule, it is recommended to begin fit analysis by looking at Outfit before Infit, and MNSQ before ZSTD. The expected value for MNSQ is approximately 1.0, and values between 0.5 and 1.5 are considered productive for measurement [26]. If the MNSQ value is beyond this range, ZSTD must be checked – ZSTD values of 2.0 or more indicate statistically significant model misfit [26]. If misfitting items or individuals are found, an iterative process proposed by Linacre (2010) may be used to address these issues [27]. Analysis begins by deleting the “really really bad” items and individuals, and then the analysis is run again. In the next step, the “really bad” items and individuals are further deleted, and the analysis is run again while results are compared to the previous step. The process continues until the most adequate statistics are obtained.

Unidimensionality was examined with Principal Component analysis (PCA) of the residuals. In unidimensional measures, it is expected that the observed variance explained by the measures roughly matches the expected variance in the model. In addition, PCA analyses the components in the correlation matrix of the residuals (called contrasts). The “first contrast” is the component that explains the largest possible amount of variance in the residuals [26]. If the unexplained variance found in the first contrast is up to 2.0 Eigenvalue, the biggest possible secondary dimension has the strength of less than 2 items. The decision to consider a measure as unidimensional or multidimensional is usually made by the researcher according to the purposes of the test. However, unexplained variances in the first contrast greater than 2.0 Eigenvalue may indicate the presence of a second dimension [26].

Reliability was evaluated using the indices provided by Winsteps: person separation index, person reliability, item separation, and item reliability. The separation indices give an estimate of the spread of items or individuals along the continuum of ability and reflect the number of distinct strata in which the sample or items can be divided [28]. The reliability reports how reproducible the person and item measure orders (i.e., their locations on the continuum) are [26]. A person separation index of 1.5 or a person reliability coefficient of 0.7 represent an acceptable level of separation and is considered the minimum required to divide the sample into two distinct strata (i.e., low and high ability) [29], while a person separation index of 2.0 and a person reliability of 0.8 represent a good level of separation and are considered the minimum preferable values [26]. Item separation index and item reliability are interpreted using the same criteria. According to Rasch guidelines, if the item reliability and separation are below the required values, a bigger sample is necessary; if the person reliability and separation are below the required values, the test needs more items [26].

Differential Item Functioning (DIF) was explored in two sub-groups of individuals categorized by gender (men and women) and duration of symptoms (acute or chronic – defined as more than 6 months of symptom duration). The presence of noticeable DIF was defined by two criteria: 1) DIF contrast >0.5 logits, and 2) significant enough not to have occurred by chance (*t* > 2.0) [26].

P-Scale item difficulty and person ability were plotted graphically in a person-item map. The person-item maps (also called Wright Maps) allow the visual analysis of the relationship between the measures of individuals and items. The use of these maps assists in the assessment of positive and negative issues, such as item redundancy (i.e., items at the same difficulty level), trait gaps (that may indicate the need of more items to fill the gaps), ordering of items matching the prediction of the test author or users (i.e., construct validity), and targeting between the items and sample (i.e., whether item difficulty range matches the sample ability range) [21].

### Sample size

The sample size (302 participants) was adequate for Rasch analysis. According to parameters defined by Linacre (1994), a sample size of 243 respondents may be sufficient to provide 99% confidence of the person estimates being within ±0.5 logits [30].

## Results

### Participants

The participants’ socio-demographic and health condition characteristics, as well as the P-Scale items on which the participants reported problems, are presented in Table 1. Among the 302 participants, the mean age was 45.5 years (SD = 14.4), 88% were up to 59 years old, 52% were male, and 44% were married. The mean years of education was 7.6 (SD = 4.1) and 54% were on sick leave. Regarding health conditions, 86% had orthopaedic conditions, and the mean duration of the symptoms was 18.3 months (SD = 37.5); additionally, 52% had chronic symptoms (i.e., more than 6 months of duration).

**Table 1:** Participants’ socio-demographic / health condition characteristics (*n* = 302)

Age (years)	Mean: 45.5 (SD: 14.4; Range: 19–82)
Gender	
Male	52%
Marital status	
Single	35%
Married	44%
Divorced	15%
Widower	6%
Education (years)	Mean: 7.6 (SD: 4.1; Range: 0–21)
Work status	
Employed – working	25%
Employed – sick leave	54%
Unemployed	8%
Retired	13%
Type of health condition	
Orthopedic	86%
Neurologic	14%
Duration of the symptoms (months)	Mean: 18.3 (SD: 37.5; Range: 0–240)
Duration of the symptoms	
Acute	48% (up to 6 months)
Chronic	52% (more than 6 months)

Eighty-three percent of the participants reported they had a problem participating in at least one of the situations described by the P-Scale items (Table 2). More than half of the sample reported having participation problems on the items related to paid work: a problem on item *N2 – Work as hard as your peers do* was reported by 52% of the participants, and a problem on item *N1 – Equal opportunity as your peers to find a job* was reported by 51% of the sample. *N14 Household work*, *N12 – Move around inside/outside house/village/neighbourhood*, and *N4 – Visit places outside village/neighbourhood* were the next most frequent items on which the participants reported problems (41%, 39%, and 37% respectively).

**Table 2:** Percentage of participation problems reported by the participants on the P-Scale (n = 302)

Reported participation problems in at least one P-Scale item	83%
Reported participation problems in each P-Scale item	
N1 Opportunity to find work	51%
N2 Work as hard	52%
N3 Contribute economically to household	27%
N4 Visit places outside village/neighborhood	37%
N5 Take part in festivals and rituals	13%
N6 Take part in casual recreational/social activities	34%
N7 Socially active	22%
N8 Same respect in community	10%
N9 Opportunity to take care of yourself	26%
N11 Visit other people in community	21%
N12 Move around inside/outside house/village/neighborhood	39%
N13 Visit public places in village neighborhood	14%
N14 Household work	41%
N15 Opinion count in family discussions	12%
N16 Help other people	10%
N17 Comfortable meeting new people	14%
N18 Confident to learn new things	15%

### Rasch analysis

The first step of the Rasch analysis was to check if the P-Scale rating scale was being used in the intended way, according to the guidelines proposed by Linacre (2002) [31]. However, in this dataset, nine items (N5, N7, N8, N9, N13, N15, N16, N17 and N18) did not reach the 10 observations per category, as suggested by the guidelines. Therefore, in order to get more stable item difficulty estimates, we decided to collapse the categories to a dichotomous format. The original categories 0 – “Yes” and “Irrelevant, I don’t want to, I don’t have to” and 1 – “No problem” were collapsed in the dichotomous format as 1 – “No problem to participate”, while the original categories 2 – “Small”, 3 – “Medium”, and 5 – “Large” were collapsed in the dichotomous format as 0 – “Problem to participate”.

#### Item and person fit

In the first Rasch analysis, we found two items of the P-Scale showing misfit: Item 15 and Item 8. To correct these misfit issues, we first removed only Item 15, and then, we removed the 21 misfitting individuals identified in the analysis (Outfit MNSQ exceeding 2.0). After these deletions, all items showed good Infit statistics (Table 3). A descriptive analysis of the 21 misfitting individuals did not identify relevant differences between them and the entire sample.

**Table 3:** Item fit statistics for the 17 P-Scale items from the Rasch Analysis

P-Scale Items		First Rasch Analysis (n = 302)				Final Rasch Analysis (*n* = 281)			
		Infit Statistics		Outfit Statistics		Infit Statistics		Outfit Statistics	
		MNSQ	ZSTD	MNSQ	ZSTD	MNSQ	ZSTD	MNSQ	ZSTD
N1 Opportunity to find work		1.01	0.2	0.96	−0.3	1.02	0.3	0.97	−0.2
N2 Work as hard		0.98	−0.3	0.89	−0.9	1.00	0.1	0.87	−0.8
N3 Contribute economically to household		0.89	−1.5	0.74	−2.0	0.95	−0.7	0.80	−1.4
N4 Visit places outside village/neighborhood		0.99	−0.1	0.92	−0.8	1.06	0.8	0.99	0.0
N5 Take part in festivals and rituals		0.93	−0.6	0.78	−0.8	0.91	−0.8	0.68	−1.1
N6 Take part in casual recreational/social activities		0.90	−1.5	0.94	−0.6	0.91	−1.2	0.97	−0.2
N7 Socially active		0.86	−1.9	0.74	−1.7	0.86	−1.7	0.76	−1.4
N8 Same respect in community		1.07	0.6	*1.68* ^*a*^	1.9	1.01	0.1	0.79	−0.4
N9 Opportunity to take care of yourself		0.96	−0.5	0.84	−1.2	1.03	0.4	0.94	−0.4
N11 Visit other people in community		0.82	−2.3	0.84	−1.0	0.83	−2.2	0.71	−1.7
N12 Move around inside/outside house/village/neighbourhood		0.97	−0.4	0.98	−0.2	1.01	0.2	1.02	0.2
N13 Visit public places in village neighborhood		0.89	−1.0	0.73	−1.1	0.93	−0.6	0.83	−0.6
N14 Household work		1.11	1.7	1.25	2.4	1.16	2.2	1.40	3.3
N15 Opinion count in family discussions		1.25	2.0	*2.03* ^*a*^	3.0	–	–	–	–
N16 Help other people		1.06	0.5	0.84	−0.4	1.07	0.5	0.72	−0.8
N17 Comfortable meeting new people		1.27	2.4	1.31	1.2	1.33	2.9	1.42	1.5
N18 Confident to learn new things		1.06	0.6	1.40	1.6	1.03	0.3	0.97	0.0
	Mean	1.00	−0.1	1.05	−0.1	1.01	0.0	0.93	−0.3
	SD	0.12	1.3	0.36	1.4	0.12	1.3	0.21	1.2

Note: The MNSQ acceptable limits to productive measurement were 0.5–1.5. Values beyond these limits are considered misfitting. *MNSQ* Mean Square, *ZSTD* Z Standardized Statistic

^a^Items showing misfit

#### Dimensionality

The PCA of the residuals was conducted to examine unidimensionality and supported the existence of only one Rasch dimension. The observed variance explained by the measures was 35.5%, similar to the expected variance of 35.8% in the model. The unexplained variance in the first contrast was 7.6% (Eigenvalue: 1.9) and less than the variance explained by the items (18.1%). For the purposes of this study, these values represent acceptable evidence for unidimensionality.

#### Reliability

The person separation index was 1.51 (person reliability 0.69), considering the real non-extreme values in the sample (i.e., omitting individuals who got the minimum or the maximum values in the scale). The person separation index values indicate an acceptable level of separation, distinguishing two strata of participation ability in the sample: high participation and low participation [29]. However, these values are less than those required for a “good level” of separation, suggesting that more items may be needed in order to better distinguish between high and low ability levels. The item separation index was 6.38, and the item reliability was 0.98. These values inform that the sample was large enough to confirm the item difficulty hierarchy (i.e., construct validity) [21, 26].

#### Differential item functioning

In the DIF analysis, we found two items showing noticeable differences between men and women. Item *N2 – Work as hard your peers do* was 0.71 logits more difficult for men than for women, while item *N14 – Household work* was 0.71 logits more difficult for women than for men. When analysing DIF related to the duration of the symptoms, we also identified two items showing noticeable differences between the groups (acute vs. chronic). Item *N6 – Take part in casual recreational/social activities* was 0.73 logits more difficult for people with acute conditions, while item *N1 – Move around inside/outside house/village/neighbourhood* was 0.84 logits more difficult for people with chronic conditions. Our study aimed to examine how the structure of the P-Scale behaved when administered in this clinical sample and who attended the public health care system in Brazil. Considering that modifying the scale structure was not the purpose of this study, we hope this information will contribute to help guide the scale’s use in clinical practice and future research.

#### Item difficulty (hierarchy)

**Fig. 1:**
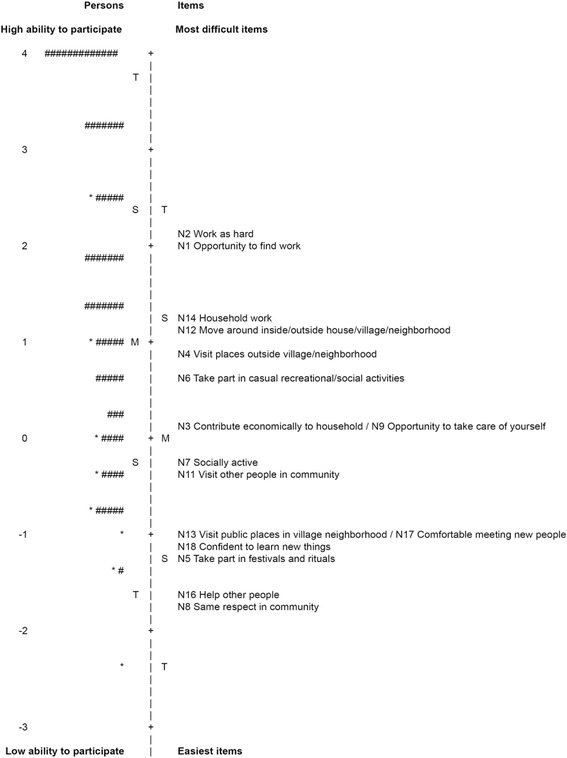
Person-Item Map of the 16 P-Scale items in the final Rasch Analysis (n = 281). Note: Each “#” = 4 persons and each “*” = 1 to 3 persons; M = Mean persons’ ability or mean items’ difficulty; S = one standard deviation; T = two standard deviations. The vertical line is a continuum representing the measures of persons’ ability (left side) and items’ difficulty (right side), plotted in logit units. The persons’ ability and items’ difficulty increase from the bottom to the top

The person-item map (Fig. 1) shows the distribution of sample measures matched to P-Scale item difficulty along the same continuum of ability levels. The items located in the bottom part of the continuum are the easiest, and the individuals located close to these items are the ones with less of an ability to participate, according to the P-Scale. On the other hand, the items located at the top part are the most difficult and the individuals located near these items have a greater ability to participate. The item difficulty ranged from item *N8 – Same respect in the community* (−1.78 logits) to *N2 – Work as hard your peers do* (2.09 logits), while the sample ability ranged from −2.41 to 4.61 logits. The items are spread on the continuum, although there are some gaps in the scale, as seen in the person-item map (Fig. 1). However, there are no items aligned with 37% of the sample, located on the top range of ability, indicating that the P-Scale may need some more difficult items to be able to assess individuals with low restriction to participation.

## Discussion

This was an initial study that investigated the psychometric properties of the P-Scale using Rasch analysis to examine whether the P-Scale may be a suitable tool to collect data regarding participation among the patients from a Brazilian public rehabilitation services network.

The participants were adults who were in treatment or seeking rehabilitation services, with diverse health conditions, socio-demographic backgrounds and functional needs, and from different services of the rehabilitation network. These sample features corresponded to the usual daily variability found in the services.

The findings are encouraging, especially when we recognize the challenges defining the construct of participation, as well as developing good measurement tools to capture it. There is consensus in the field that participation is a multifactorial construct that is related to an individual’s physical and social environments, personal factors, and health conditions [1, 3–5]. Such complexity makes participation a difficult construct to assess during a rehabilitation process, although involvement in real life activities is the final goal of most people receiving rehabilitation care. The P-Scale was found to be helpful in this particular rehabilitation context, despite some issues that should be addressed for further improvement of the scale.

Rasch analysis was performed using a modified rating scale, collapsing the original categories into a dichotomous scale since some items did not have a sufficient number of answers in all response categories [31]. This low number did not seem to be an issue related to the sample size, as the item separation index confirmed that the number of participants in our study was sufficient to test the scale items [26]. A low rate of response to some categories can occur for reasons besides sample size, such as when the respondents have difficulty distinguishing between similar categories. Further research with the P-Scale is needed to confirm whether some categories on the original rating scale are actually underused by the respondents.

After collapsing the rating scale to a dichotomous scale, we were able to perform the next steps of the analysis. In general, the P-Scale showed a good fit to the Rasch model with only one item (*N15 – Start or maintain a relationship*) showing misfit, which was thus removed in the final analysis. The low rate of misfitting items is a good indicator of unidimensionality [28]. Additionally, the dimensionality analysis performed using the Winsteps also showed that the scale could be considered unidimensional for the purposes of the Rasch analysis. Although the variance explained by the measures reached a low percentage (approximately 35%), it was closely matched to the variance expected in the model. The variance explained by the residuals in the first contrast was below 2.0 Eigenvalues, indicating the low likelihood of a second dimension [26].

Even using a modified rating scale format, the P-Scale can be useful as a screening tool for participation problems reported by patients in order to lead rehabilitation professionals in addressing such problems in the patients’ recovery process. In the analysis, the items were well spread along the continuum of difficulty, with just four items overlapping at the same measure of difficulty (*N3 – Contribute economically to household* and *N9 – Opportunity to take care of yourself*; and *N13 – Visit public places in village neighbourhood* and *N17 – Comfortable meeting new people*). Thus, the items were able to show where most of the patients were located on this difficulty continuum, according to their ability to participate. However, as seen in the person-item map, there are some gaps between the items. In the same way, there were no items covering the top of the continuum, in which the best performing patients were located (approximately 37% of the sample).

The gaps and lack of items to represent the patients with higher participation abilities may explain the person separation index found in this study (1.51). This index is influenced by factors such as the length of the scale, number of categories per item, and the match between the items and the ability of the respondents (i.e., sample-item targeting) [26]. Although the person separation index found in this study indicates that the items were enough to discriminate the sample in two groups (low and high ability to participate), a higher index value is desirable for more sensitivity and improved identification of different levels of ability among the respondents [21, 26]. Because the P-Scale was first developed to assess the participation of people with disabilities related to stigma [10], some items (e.g., *N8 – Same respect in the community*) seem to be more useful for individuals with severe restrictions than for those with low or moderate restrictions. In this sense, it might be useful to expand the scale by adding some items that could better identify participation restrictions among individuals with better functioning.

In an effort to make the P-Scale more suitable to people with different health conditions and disabilities, the scale authors removed one item from the former version [10] – *N16 – In home, are the eating utensils you use kept with those used by the rest of the household?* – because this item was considered more appropriate for individuals with infectious health conditions, such as Leprosy. It is expected that the new item added in the latest version [24]: *N10 – Do you have the same opportunities as your peers to start or maintain a long-term relationship with a life partner?* will be more relevant for diverse health conditions. However, because only a portion of the patients in the present study sample answered the latest P-Scale version, we were not able to include this new item on the analysis. Thus, it would be valuable if future research include this item as it potentially can contribute to fill some of the gaps found on the continuum.

The analysis of DIF demonstrated that item difficulty varied between men and women in two items (*N2 – Work as hard your peers do* and *N14 – Household work*), while two other items (*N6 – Take part in casual recreational/social activities* and *N12 – Move around inside/outside house/village/neighbourhood*) showed differences between people with acute symptoms and people with chronic symptoms. These variations may be attributed to different engagements of each group in the situations described by the items due to cultural factors. However, before suggesting any changes on these items, further investigation should be carried out to clarify why the respondents answered differently to those situations and whether DIF has a significant impact on the overall score.

## Conclusions

The major contributions of this study are to demonstrate the usefulness of the P-Scale in rehabilitation services networks and provide useful information to further improve the tool. Having the P-Scale items ordered in a logit scale would make it possible to create interval-level summary scores that reflect the relative difficulty of each item to more accurately assess the participation engagement among the patients in rehabilitation care. A better measure would also allow for comparison of patients’ participation ability and needs for different professionals across rehabilitation services and diverse health conditions. This information could be used to guide planning and investment in the rehabilitation network, such as the need to hire additional rehabilitation professionals, improvement or creation of services, or the development of specific rehabilitation programmes for the rehabilitation network.

## Abbreviations

CTT: Classical Test Theory
DIF: Differential Item Functioning
ICF: International Classification of Functioning, Disability and Health
MNSQ: Mean Square
PCA: Principal Component Analysis
P-Scale: Participation Scale
SD: Standard Deviation
WHO: World Health Organization
ZSTD: Z-Standardized Scores
